# A Common Neurological Presentation With an Unusual Etiology: Dense Hemiplegia Due to Pneumococcal Meningitis

**DOI:** 10.7759/cureus.26624

**Published:** 2022-07-07

**Authors:** Rohan J Verghese, Kandan Balamurugesan, Abdoul Hamide, Anand Kumar

**Affiliations:** 1 Department of Medicine, Jawaharlal Institute of Postgraduate Medical Education and Research, Puducherry, IND

**Keywords:** complicated meningitis, focal neurological deficit, acute hemiplegia, pneumococcal meningitis, brain abscess

## Abstract

Acute hemiplegia is a common neurological presentation that usually occurs due to a cerebrovascular accident. A similar presentation may also be seen in several other conditions such as postictal (Todd’s) paralysis, hemiplegic migraine, brain abscess, and extradural or subdural hemorrhage. We present the case of a 32-year-old South Indian female who was brought to the emergency department with acute hemiplegia and decreased responsiveness for one day. She was provisionally diagnosed with an ischemic stroke at presentation; however, contrast-enhanced computed tomography (CECT) of the brain with CT angiography and venography revealed no focal lesions or filling defects. CSF examination showed gram-positive cocci in pairs, concerning brain abscess. Magnetic resonance imaging (MRI) of the brain was suggestive of multiple evolving abscesses in the right frontal and parietal lobes. Her hemiplegia was attributed to the abscess, and she was given six weeks of intravenous (IV) antibiotics, after which she recovered completely. Maintaining a high index of clinical suspicion enabled the correct diagnosis in a patient who did not have any typical features of acute meningitis.

## Introduction

Acute hemiplegia is a common neurological presentation seen in emergency departments throughout the world. It is typically caused by a cerebrovascular accident and usually results in the activation of the stroke management protocol. However, previous studies have shown that up to a quarter of patients presenting with an acute stroke may have “mimics” such as postictal (Todd’s) paralysis, hemiplegic migraine, and extradural or subdural hemorrhage [1].

Brain abscesses are another important cause of focal neurological deficits, although they typically present with high-grade fever, altered consciousness, and meningeal signs before the onset of neurological deficits [2]. Rarely, however, focal deficits may precede the development of fever in a patient with complicated meningitis, thus presenting similarly to a stroke [3]. This case demonstrates the need to consider brain abscess among the differential diagnoses in a patient with acute hemiplegia.

## Case presentation

A 32-year-old female, a manual laborer by occupation with no previous comorbidities, was brought to the emergency department with a history of diminished responsiveness for 12 hours. She had complained of headaches and blurred vision for two days before arrival but did not have any history of fever. She was unresponsive to commands, and her attendees also reported a lack of movements of her left upper and lower limbs.

At the initial presentation, she was unresponsive to commands, and her Glasgow Coma Scale (GCS) score was 9/15. Her heart rate was 56/minute, and her blood pressure was 160/90 mmHg. On sternal pressure, she was able to localize pain with her right upper limb but had no movement in her left upper and lower limbs. Her pupils were equal and reactive to light, and fundus examination revealed bilateral early papilledema. She was also noted to have neck stiffness but had no other signs of meningeal irritation.

**Figure 1:**
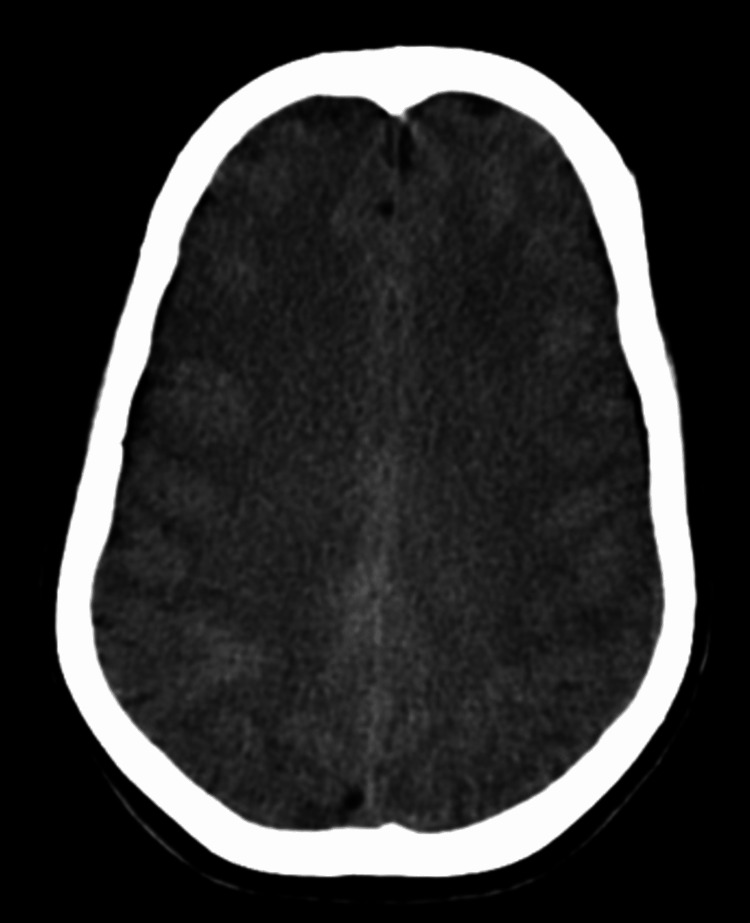
Non-contrast CT of the brain showing diffuse cerebral edema.

**Figure 2:**
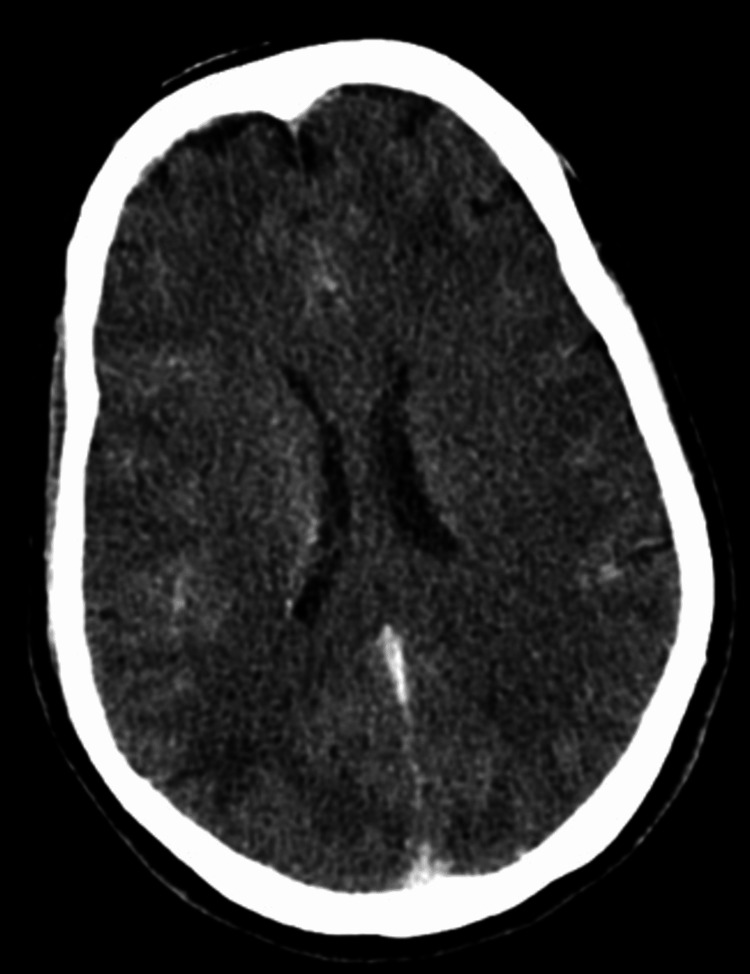
Contrast-enhanced CT of the brain showing no focal lesions or cortical venous thrombosis.

Due to the patient’s acute presentation, a hemorrhagic cerebrovascular accident or cerebral venous thrombosis was considered the initial diagnosis. A non-contrast brain computed tomography (CT) was done, which showed no signs of an infarct or hemorrhage (Figure 1). Contrast-enhanced CT (CECT) of the brain with CT venogram showed no focal lesions or contrast filling defects (Figure 2).

Her complete blood count revealed neutrophilic leukocytosis with a total leukocyte count (TLC) of 15,310/mm^3^. This led us to consider an infectious cause of hemiplegia. A lumbar puncture was done, and CSF analysis showed 1,420 WBCs with a neutrophilic predominance (95%) and low CSF glucose (1 mg/dL). CSF gram stain showed gram-positive cocci in pairs. Based on this, she was empirically started on intravenous ceftriaxone and vancomycin.

**Figure 3:**
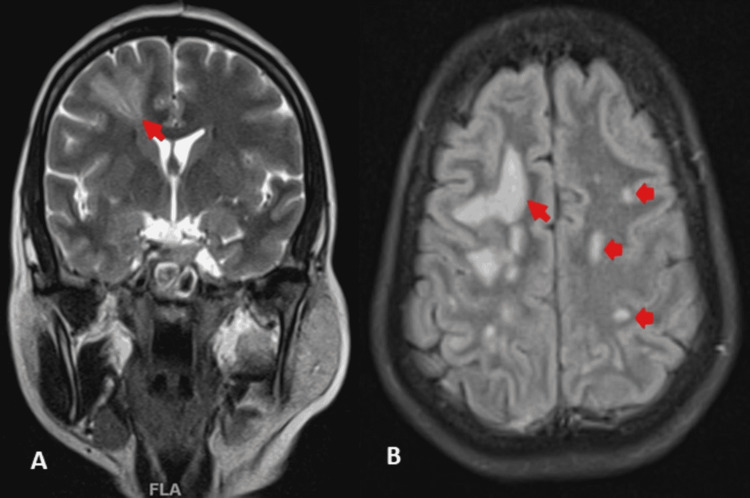
A: Coronal T2 FLAIR MRI of the brain showing T2 hyperintensity predominantly in the right frontal lobe (red arrow), suggestive of a brain abscess. B: Axial T2 FLAIR MRI of the brain showing the same lesion with multiple similar lesions in both cerebral hemispheres.

**Figure 4:**
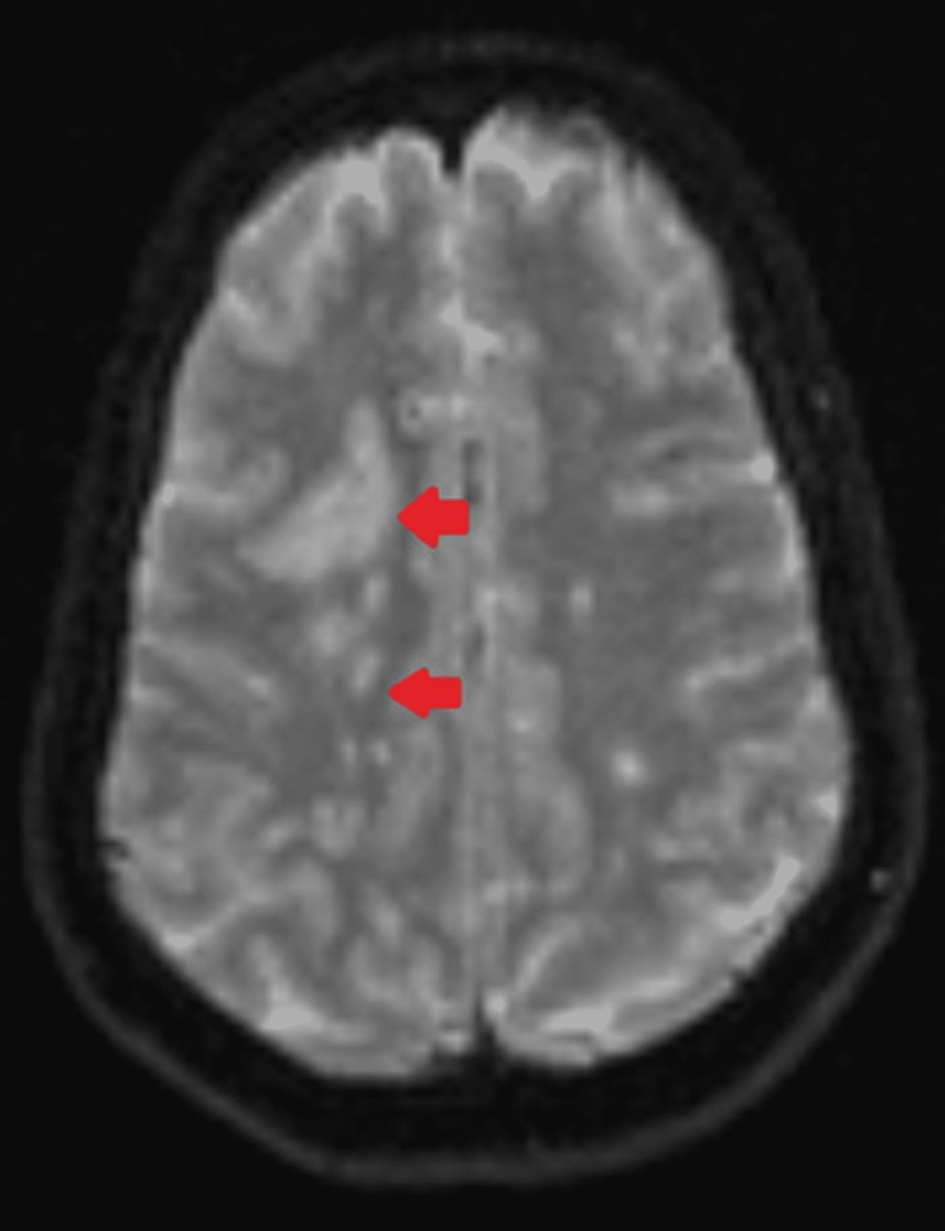
Diffusion-weighted MRI showing diffusion restriction in the lesions (red arrows).

**Figure 5:**
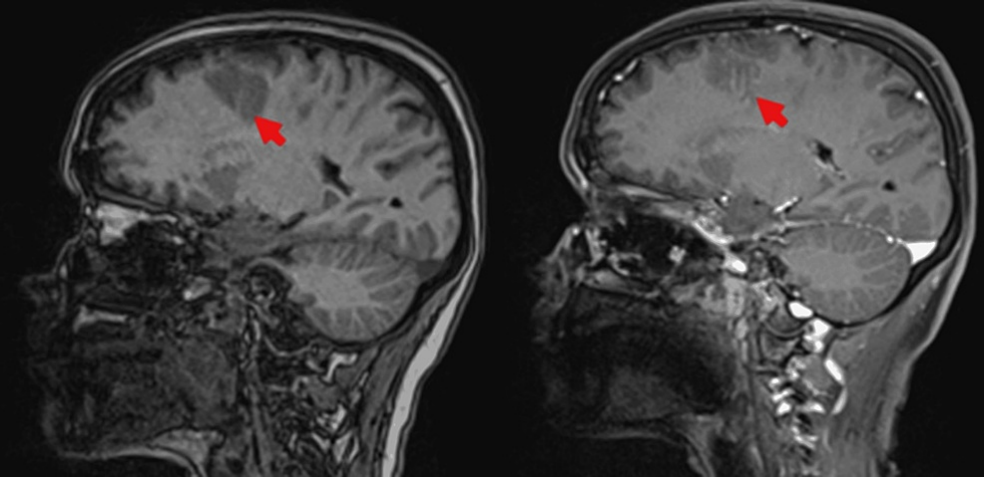
Sagittal T1-weighted image with contrast showing contrast enhancement of the lesions.

**Figure 6:**
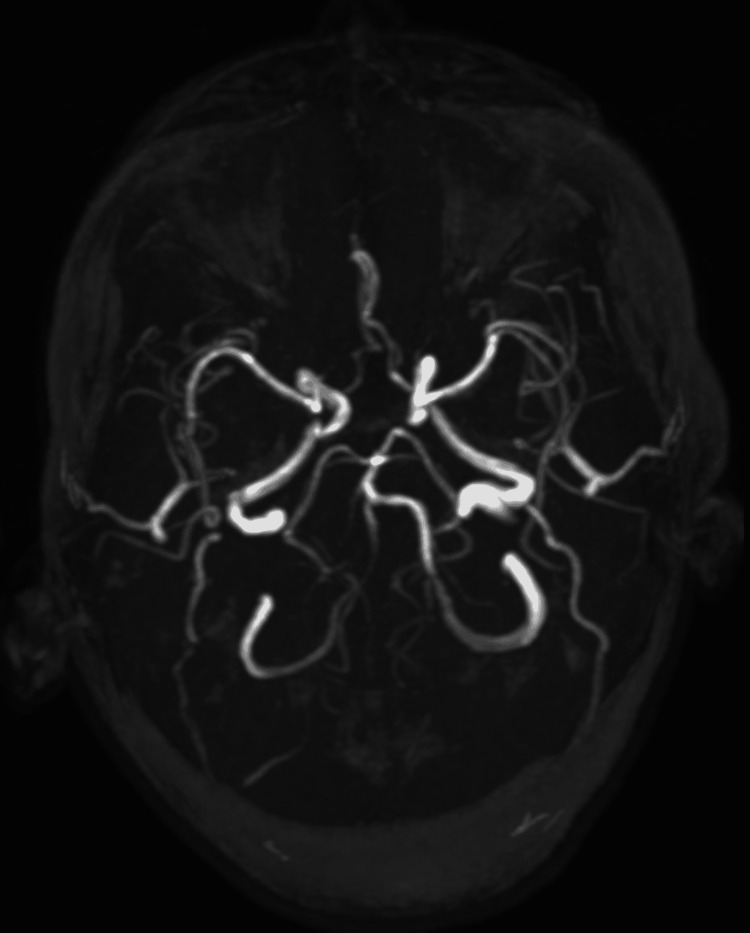
MR angiography showing normal vessels.

A magnetic resonance imaging (MRI) of the brain was done, which showed multiple T2 hyperintense lesions in both hemispheres but primarily in the right frontal and parietal lobes, the largest of which was 4 × 2 × 2 cm (Figure 3). These lesions showed diffusion restriction (Figure 4), but contrast imaging revealed contrast enhancement against the diagnosis of an infarct (Figure 5). MR angiography was normal (Figure 6). These features were consistent with a diagnosis of evolving brain abscesses. CT and MRI images were reviewed, and no cranial defects were noted. Detailed ear, nose, and throat examination revealed no signs of sinusitis or otitis media, and echocardiography showed no vegetations.

Blood and CSF cultures grew *Streptococcus pneumoniae*, which was sensitive to vancomycin. Ceftriaxone was stopped, and vancomycin was continued based on this sensitivity report. She began to show improvement on day 2 of vancomycin, and her sensorium returned to normal on the third day. Physiotherapy was initiated once the sensorium normalized and was continued for the duration of the hospital stay. Power in her left limbs began to improve by day 8 of admission. Repeat blood and CSF cultures done after one week were sterile. She was given intravenous vancomycin for six weeks as per guidelines for brain abscess [4] and had no residual deficits at discharge.

The patient was reviewed one month after discharge and remained asymptomatic with complete neurological recovery. She was able to return to work as a farm laborer.

## Discussion

Brain abscess is an uncommon life-threatening neurological condition with a high mortality rate of up to 36% [4]. It typically presents with a triad of fever, headache, and focal deficits. However, on occasion, brain abscesses can present without fever. A 15-year survey by Helweg-Larsen et al. in Denmark showed that up to 42% of pyogenic brain abscesses can present without fever [5]. Several other case series showed that fever occurs in less than 50% of pyogenic abscesses [6-8]. In the Danish survey population, lack of fever was reported as a common cause of delay in diagnosis [5]. In fact, in 10 of the cases in the series, the initial CT was interpreted as consistent with a stroke or brain tumor due to low suspicion before the imaging. Similarly, in our patient, the absence of fever led us first to consider a vascular etiology and investigate for the same, which caused a delay in the institution of appropriate antibiotics.

The differentiation between brain abscess and tumor or infarct by CT is often not straightforward. Frequently, CT cannot pick up small abscesses, with the diagnosis being reached after an MRI. We faced a similar situation with our patient, where the initial interpretation of the contrast CT reported the absence of focal lesions. The lesions were only picked up later by an MRI, which was initially unavailable. Such unavailability of access to urgent MRI was one of the causes of delay in diagnosis listed in the Danish survey population [5].

*Streptococcus pneumoniae* is the leading cause of meningitis in adults. Some CNS complications described in pneumococcal meningitis include seizures, cerebral infarcts, hydrocephalus, and hearing loss [9]. Brain abscess, however, is a rare complication of pneumococcal meningitis, and *Streptococcus pneumoniae* accounts for only 2% of all bacterial brain abscesses [10]. In fact, out of 2,216 culture-positive brain abscesses reported in India from 2010 to 2019, only Sarmast et al. reported the isolation of *Streptococcus pneumoniae* in 22 of their patients [4].

Such brain abscesses are usually seen in association with sinusitis or otitis media. Mathern and Calestino [3] described a case similar to ours, where a patient presented with symptoms and signs of a middle cerebral artery stroke. A rapid drop in sensorium and the development of high-grade fever in the hospital lead them to perform a lumbar puncture and diagnose pneumococcal meningitis. In their case, the patient was documented to have mastoiditis by CT, and imaging showed no evidence of a brain abscess. In contrast, we did not detect any signs of mastoiditis or otitis media in our patient. Pneumococcal brain abscess has also been described in the setting of infective endocarditis and pneumonia by Vandenbos et al. [11]. This was also in contrast to our patient, who had no other organ involvement despite having a bloodstream infection.

Several aspects of the management of brain abscesses remain controversial, including the need for surgery and optimal surgical approaches, the type and length of antibiotic treatment, and the need for monitoring during treatment. No randomized controlled prospective trials are available for the same. British guidelines for managing brain abscesses recommend a 4- to 6-week course of antibiotics with surgical drainage [12]. However, a previous case report by Belodu et al. described the complete resolution of a pneumococcal brain abscess with an extended duration of oral antibiotic therapy [13]. Similarly, our patient improved well with medical management, obviating the need for surgery.

## Conclusions

While evaluating a patient with acute hemiplegia, unusual causes such as complicated meningitis, cortical venous thrombosis, and Todd’s paralysis also need to be considered. Brain abscess is a rare complication of pneumococcal meningitis and may present as an acute-onset focal neurological deficit. Early diagnosis and administration of appropriate antibiotics for the optimal duration can lead to complete neurological recovery in these patients.
